# Endocannabinoids Generated by Ca^2+^ or by Metabotropic Glutamate Receptors Appear to Arise from Different Pools of Diacylglycerol Lipase

**DOI:** 10.1371/journal.pone.0016305

**Published:** 2011-01-28

**Authors:** Longhua Zhang, Meina Wang, Tiziana Bisogno, Vincenzo Di Marzo, Bradley E. Alger

**Affiliations:** 1 Department of Physiology, University of Maryland School of Medicine, Baltimore, Maryland, United States of America; 2 Department of Psychiatry, University of Maryland School of Medicine, Baltimore, Maryland, United States of America; 3 Program in Neuroscience, University of Maryland School of Medicine, Baltimore, Maryland, United States of America; 4 ECB Research Group, Institute of Biomolecular Chemistry, National Research Council, Pozzuoli, Italy; The Research Center of Neurobiology-Neurophysiology of Marseille, France

## Abstract

The identity and subcellular sources of endocannabinoids (eCBs) will shape their ability to affect synaptic transmission and, ultimately, behavior. Recent discoveries support the conclusion that 2-arachidonoyl glycerol, 2-AG, is the major signaling eCB, however, some important issues remain open. 2-AG can be synthesized by a mechanism that is strictly Ca^2+^-dependent, and another that is initiated by G-protein coupled receptors (GPCRs) and facilitated by Ca^2+^. An important question is whether or not the 2-AG in these cases is synthesized by the same pool of diacylglycerol lipase alpha (DAGLα). Using whole-cell voltage-clamp techniques in CA1 pyramidal cells in acute *in vitro* rat hippocampal slices, we investigated two mechanistically distinct eCB-mediated responses to address this issue. We now report that pharmacological inhibitors of DGLα have quantitatively different effects on eCB-mediated responses triggered by different stimuli, suggesting that functional, and perhaps physical, distinctions among pools of DAGLα exist.

## Introduction

The cannabinoid system affects behavior and regulates many synaptic functions. There are two major endogenous ligands for CB1R (the main cannabinoid receptor in the brain): the eCBs N-arachidonoyl-ethanolamine (anandamide [1]) and 2-arachidonoyl-glycerol (2-AG) [2], [3], [4]. Anandamide and 2-AG have different synthetic and degradative pathways, and the eCB-dependent regulation of neuronal communication will be determined by the identity and subcellular sources of the eCB involved. Evidence is converging on the conclusion that 2-AG is the primary phasic signaling eCB at numerous synapses in the brain [5], whereas anandamide may regulate tonic eCB actions [6]. Strong support for the former inference comes from recent molecular genetic studies in which the primary synthetic enzyme for 2-AG, DAGLα, was knocked out in lines of mutant mice [7], [8], causing a reduction of ∼80% in basal 2-AG levels. Purely Ca^2+^-dependent eCB signaling – depolarization-induced suppression of inhibition, DSI [9], [10], and excitation, DSE [11] – and eCB signaling mediated by GPCRs, including group I metabotropic glutamate receptors (mGluRs), i.e., (eCB_mGluR_) [12], [13] were essentially abolished by DAGLα deletion. Yet, additional issues remain unresolved. For example, it is not known if the same DAGLα source (pool) provides 2-AG for both DSI and eCB_mGluR_.

eCBs mediate different forms of synaptic plasticity [14], hence knowledge of the cellular source(s) of eCBs is an important issue, yet one that cannot be addressed with a global knock-out strategy. Accordingly, we have taken a pharmacological approach, using two DAGL inhibitors to determine whether the pools of Ca^2+^ - and mGluR-dependent of 2-AG are distinguishable. If eCB responses to both stimuli were equally sensitive to the inhibitors, it would argue that the sources of 2-AG are the same, whereas marked differences in sensitivity would indicate that on a functional, and perhaps physical, level they differ. We report that the DAGL that mediates hippocampal DSI and eCB_mGluR_, can be functionally separated into two pools. Understanding the differences in subcellular regulation of 2-AG may lead to new modes for controlling eCB actions.

## Results

**Figure 1:**
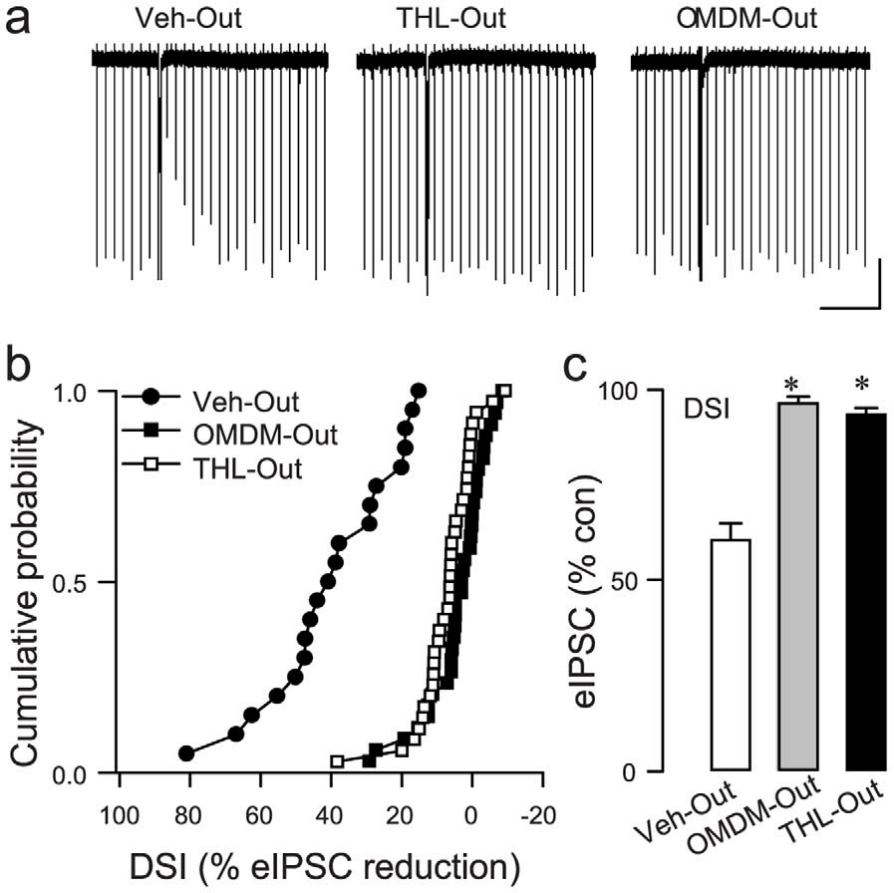
External application of DAGL inhibitors blocks DSI. (a) Representative DSI trial. Downward deflections are eIPSCs evoked at 4-s intervals; DSI was evoked by a 3-s voltage step to 0 mV from the holding potential of -70 mV; depression of eIPSCs after a step is the period of DSI (see text). Scale: 24 s/200 pA. (b) Bath application of OMDM-188 (5 µM) or THL (10 µM) essentially abolished DSI; K-S tests, p<0.01. Note: values <0 represent eIPSCs that were greater than baseline amplitudes, not enhanced DSI. (c) Group data. ^*^ p<0.001, one way ANOVA on ranks. Vehicle, n = 20; OMDM-188, n = 34; THL, n = 35.

**Figure 2:**
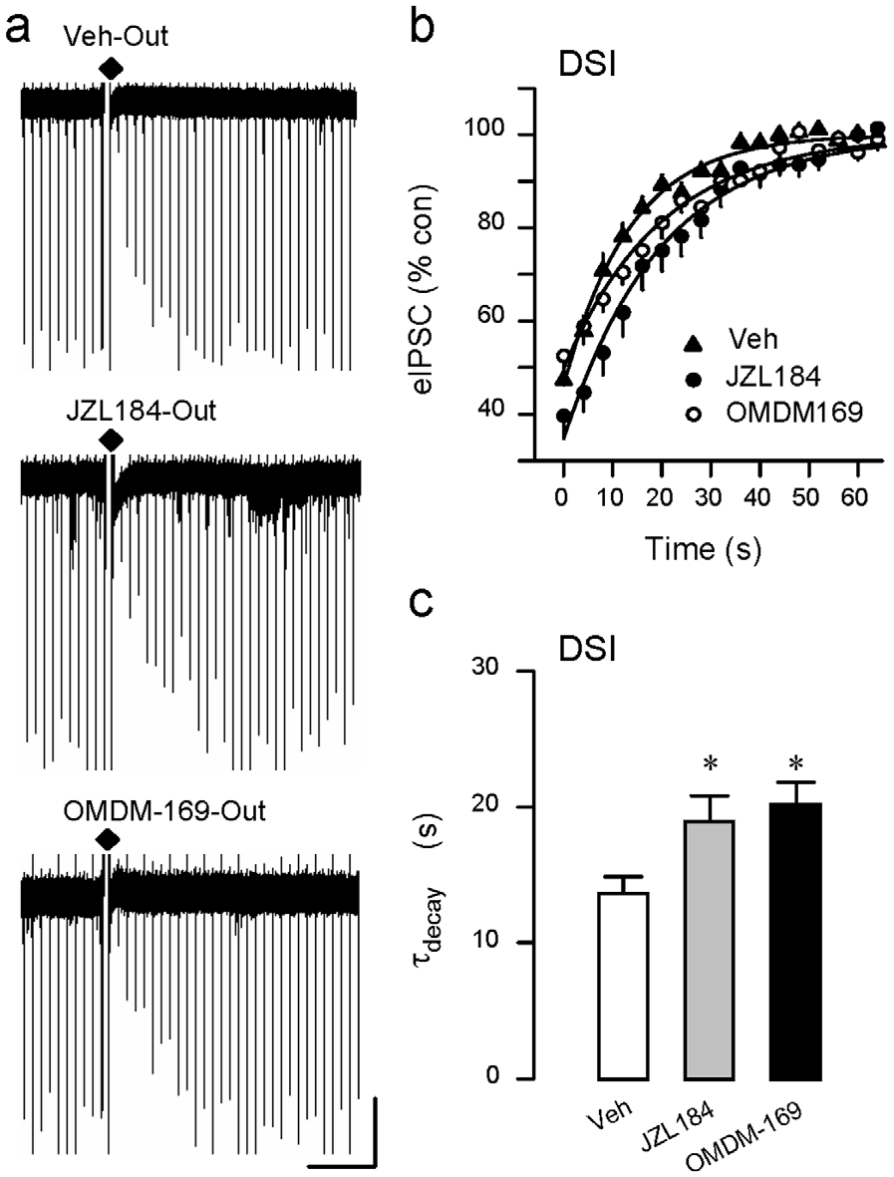
Antagonists of the primary catabolic enzyme for 2-AG, monoacylglycerol lipase (MAGL), prolong DSI. Diamonds indicate delivery of DSI-inducing voltage steps. Scale: 30 s/150 pA. (a) Bath application of MAGL inhibitors, JZL184 (1 µM) or OMDM-169 (2 µM), prolong DSI. (b) Group data showing recovery DSI in the presence of DMSO (Veh), JZL184, or OMDM-169. The DSI-inducing voltage step ended 1 s prior to time 0. The solid lines are best fitting single-exponential functions; the time constants of these functions were taken as the decay time constants (τ_decay_) of DSI. (c) Group data showing increases in τ_decay_ of DSI in the indicated conditions. When applied for 40–120 min, JZL184 or OMDM-169, prolonged DSI; τ_decay_ was increased by ∼40% (DMSO: 13.9±1.1 s, n = 21; JZL184: 19.2±1.7 s, n = 15; OMDM-169: 20.4±1.6 s, n = 15; p<0.01, one way ANOVA).

While recent molecular biological evidence supports the conclusion that 2-AG is the signaling eCB, pharmacological tools can be useful in teasing apart subtle features of the DAGLα/2-AG system that are not revealed by constitutive knock-out strategies. To test the hypothesis that both DSI and eCB_mGluR_ are mediated by the same source of 2-AG, we began by bath-applying DAGL inhibitors to voltage-clamped hippocampal CA1 cells in acute slices in which inhibitory post-synaptic currents (IPSCs) were pharmacologically isolated (see Methods). External application of the selective and potent inhibitor, OMDM-188 [15], 5 µM, or the less-selective inhibitor, tetrahydrolipstatin (THL), 10 µM, abolished DSI of evoked IPSCs (eIPSCs). As a percentage of baseline (100%) level, eIPSCs in the various conditions were: Vehicle: 60.2±4.0%, n = 20; OMDM-188: 95.7±1.5%, n = 34; THL: 92.8±1.4%, n = 35 (Fig. 1). We also tested two inhibitors of the 2-AG degradative enzyme, monoglyceride lipase, as these inhibitors do not affect anandamide. Both JZL 184 [16], 1 µM, and OMDM-169 [15], 2 µM, significantly prolonged τ_decay_ of DSI (cf [17]), thus providing an independent cross-check on the hypothesis that DSI is mediated by 2-AG (Fig. 2).

**Figure 3:**
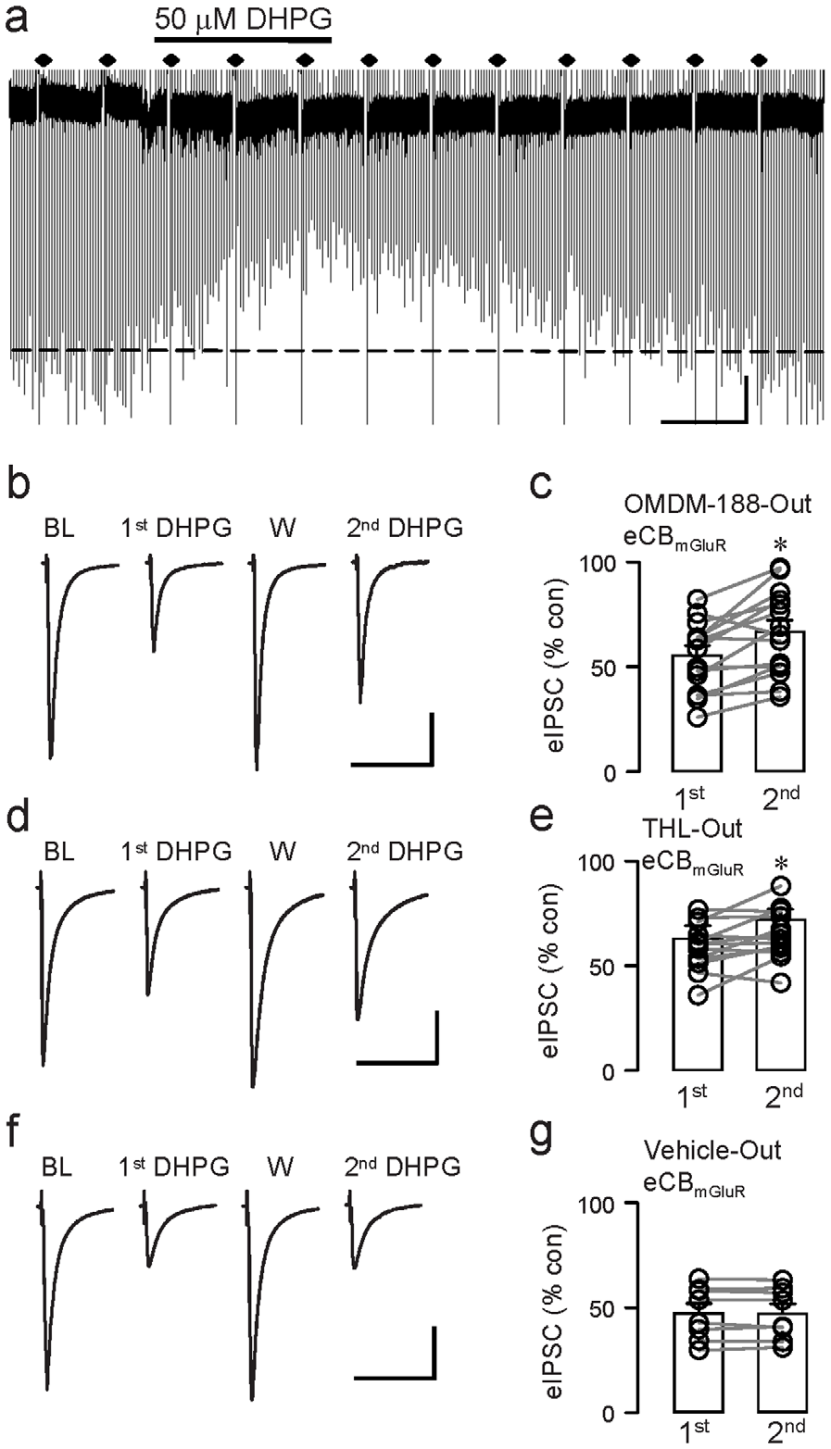
External application of DAGL inhibitors has minimal effects on eCB_mGluR_. (a) Sample trace showing eIPSCs (downward deflections) and DSI trials (diamonds) in external OMDM-188, 5 µM. Note DSI is abolished despite continued suppression of eIPSCs by DHPG. (b) Sample eIPSCs (each trace is the mean of 3) from the same cell in external OMDM-188. BL denotes the baseline response, and W, the response obtained after washing out DHPG. DHPG was applied twice at 20-min intervals starting ∼15–20 min after break-in. (d) As in (b), with THL in the saline. (f) As in (b), with vehicle only in the saline. (c)(e)(g) Group data for experiments in (b), (d), and (f), respectively (paired-t-tests). OMDM-188-Out, n = 15, p<0.01; THL-Out, n = 13, p<0.05; Vehicle-Out, n = 8, p>0.5. Scale: 20 ms/200 pA.

Unlike DSI, eCB_mGluR_-dependent eIPSC suppression (e.g., Fig. 3a) was highly resistant to external application of DAGL inhibitors. Responses to initial applications of DHPG were not different (p>0.05) whether the slices were treated with vehicle, or DAGL inhibitors. Even when evoked by repeated 4-min bath-applications of a high concentration of the group I mGluR agonist, DHPG., 50 µM, eCB_mGluR_ was only slightly, though statistically significantly, reduced by OMDM-188 (1^st^ DHPG: eIPSC reduction to 54.4±4.3% of baseline; 2^nd^ DHPG: to 65.6±5.2% of baseline; n = 15, p<0.005, Figs. 3b, 3c) or THL (1^st^ DHPG: to 58.9±3.1% of baseline; 2^nd^ DHPG: to 64.7±3.3% of baseline; n = 13, p<0.05; Figs. 3d, 3e). In vehicle-treated cells there were no significant reductions in the responses to repeated DHPG applications: 1^st^ DHPG: to 47.5±4.5% of baseline; 2^nd^ DHPG: to 47.3±4.4% of baseline; n = 8, p>0.5, Figs. 3f, 3g). Though resistant to DAGL inhibitors, eCB_mGluR_ was strongly reduced by the CB1R antagonist, SR141716A (10 µM) (1^st^ DHPG: to 70.6±8.1% of baseline; 2^nd^ DHPG in SR141716A: to 89.9±5.2% of baseline; n = 5, p<0.05; data not shown), confirming previous reports [12], [13], that eCB_mGluR_ is CB1R-dependent.

**Figure 4:**
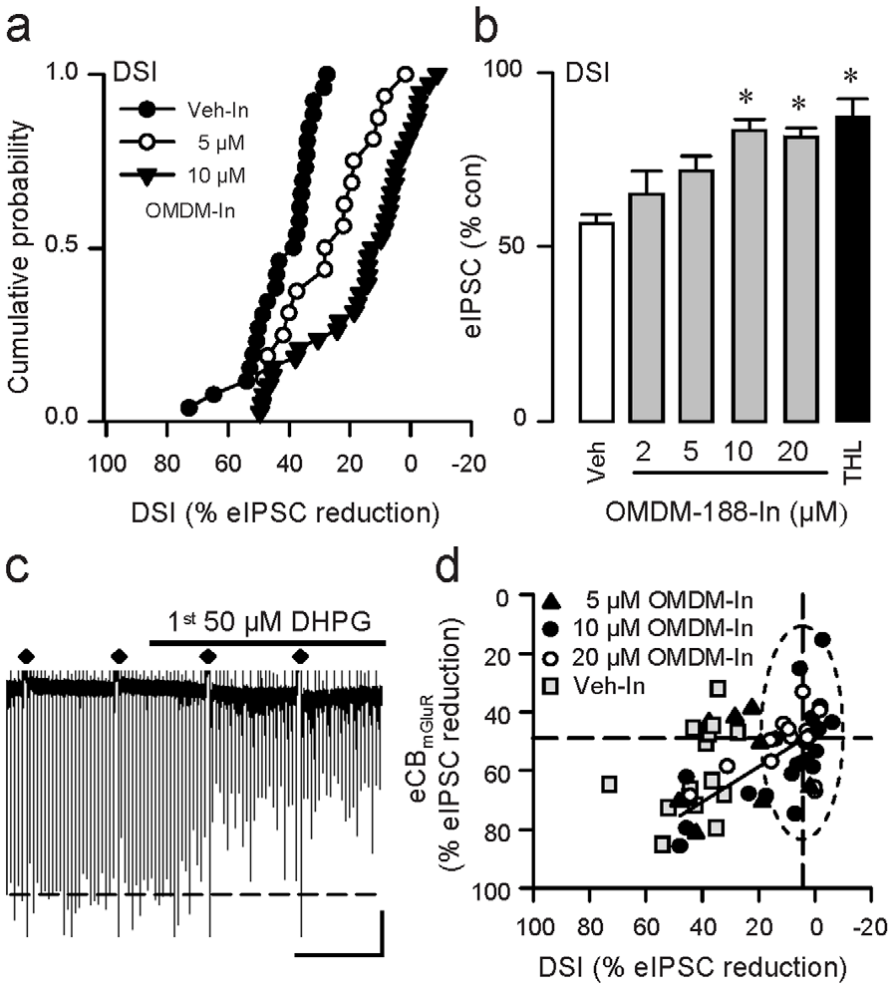
Intracellular application of DAGL inhibitors reduces DSI to a greater extent than it reduces eCB_mGluR_. (a) Intracellular infusion of OMDM-188 reduced DSI; each symbol represents averaged DSI values (3 trials) from one cell. Only data for 5 µM (n = 16) and 10 µM (n = 38) groups shown for clarity; both differ from Veh-In group, K-S test, p<0.01. (b) Group data. Includes results in (a), plus 2 µM (n = 6), 20 µM (n = 40) groups. ^*^ p<0.001, one-way ANOVA on ranks. (c) Sample continuous trace showing DSI trials and eIPSC suppression by eCB_mGluR_. Scale: 2 min/200 pA. (d) Group data for experiments as in (c) with internal 5, 10 or 20 µM OMDM-188, or Veh only. DSI and eCB_mGluR_ were measured, and eCB_mGluR_ was plotted against DSI for each cell. For the cells (n = 28/35) within dotted oval, mean eIPSC reduction from baseline by DSI is 4.3±1.06%, mean eCB_mGluR_ eIPSC reduction is 49.0±2.32%. The straight line is a linear regression fit to the data for 10 and 20 µM DHPG (see text for discussion).

Because intracellular application could conceivably be more effective on eCB_mGluR_ [18], we tested the DAGL inhibitors by applying them intracellularly via infusion through the whole-cell pipette. We observed dose-dependent reduction in DSI with internal OMDM-188 (2–20 µM) (Figs. 4a, 4b; n = 94), and similar significant reductions caused by internal application of THL (10 µM, n = 19) (Fig. 4b). Internal DAGL inhibition did not alter eCB_mGluR_ in the same way (Figs. 4c, 4d). We examined the effects of OMDM-188 in detail and found that, even in the same cells in which DSI was reduced to negligible levels (≤5% eIPSC reduction, n = 28/35 cells; see Fig. 4d, dotted oval), eCB_mGluR_ still suppressed eIPCSs by ∼50%, i.e., OMDM-188 had almost no effect on eCB_mGluR_. Internal infusion of 5 µM OMDM-188 (filled triangles in Fig. 4d), reduced either eCB_mGluR_ or DSI only slightly. Interestingly, data from the cells in which 10 or 20 µM OMDM-188 was least effective fell along a regression line around which the 5 µM data also scattered. This could mean that in these cases diffusion of 10 or 20 µM OMDM-188 out of the pipettes was incomplete, resulting in a lower-than-expected internal concentration of the drug.

**Figure 5:**
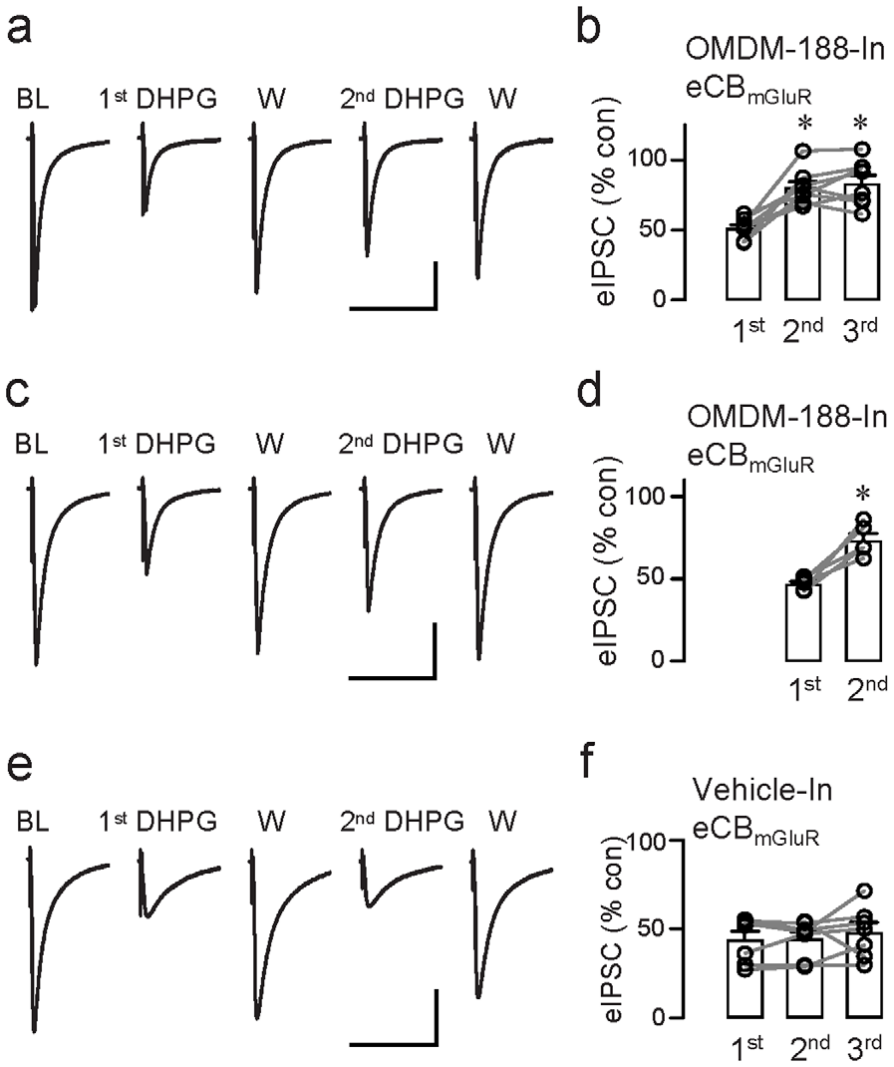
Use-dependent reduction of eCB_mGluR_ with internal DAGL inhibitor. (a) Sample eIPSCs (each trace is the mean of 3) from the same OMDM-188-loaded (20 µM in the pipette) cell in the indicated conditions. BL denotes the baseline response, and W, the response obtained after washing out DHPG. DHPG was applied 3 times at 20-min intervals starting ∼15–20 min after break-in. (c) As in (a) except that the 1^st^ DHPG application was given ∼40 min after break-in – i.e., at the same time as the 2^nd^ DHPG application in (a) – and the 2^nd^ one at 50–60 min after break-in. (e) As in (a), with vehicle only present in the internal solution. (b)(d)(f) Group data for experiments in (a), (c), and (e), respectively. ^*^p<0.001, one way repeated ANOVA; Scale: 20 ms/200 pA.

To ensure that the DAGL antagonist had an opportunity to equilibrate throughout the cells, we extended the observations and reapplied DHPG at 15–20 min intervals with OMDM-188 (10 or 20 µM) in the internal solution. Indeed, with repetitive DHPG application, eCB_mGluR_ suppression of eIPSCs diminished (1^st^ DHPG: to 51.5±2.5% of baseline; 2^nd^ DHPG: to 80.4±4.4% of baseline; 3^rd^ DHPG: to 83.5±5.6% of baseline; n = 8, p<0.001, Figs. 5a, 5b). The decrease in eCB_mGluR_ did not reflect spontaneous response ‘rundown’, because responses were stable with infusion of vehicle (DMSO or ethanol) (1^st^ DHPG: to 44.0±4.7% of baseline; 2^nd^ DHPG: to 44.4±4.0% of baseline; 3^rd^ DHPG: to 48.1±5.4% of baseline; n = 7, p>0.5; Figs. 5e, 5f).

It was not clear whether the passage of time alone accounted for the increased inhibitor efficacy, or whether suppression of eCB_mGluR_ by DAGL inhibition was use-dependent, i.e., whether it was enhanced by repetitive stimulation. To distinguish the effects of longer OMDM-188 infusions from those of repeated DHPG application, in another group of cells we delayed the 1^st^ DHPG application until 30–40 min after break-in, i.e., it was given at the same relative time after break-in as the 2^nd^ DHPG application in the original group (cf, Figs. 5b, 5d). The eCB_mGluR_ eIPSC suppression was the same in the two 1^st^ DHPG groups: (30–40 min post-break-in: to 50.5±4.3% of baseline, n = 11; 10–20 min post-break-in: to 49.5±2.5% of baseline, n = 16; n.s. p>0.1). A 2^nd^ DHPG application (i.e., 50–60 min post-break-in) given to cells receiving a delayed 1^st^ DHPG application, induced less eIPSC depression (2^nd^ DHPG: to 73.4±4.3% of baseline; 1^st^ DHPG: to 46.8±1.7% of baseline; n = 5, p<0.01; Figs. 5c, 5d). As a final check, we compared the magnitudes of the 2^nd^ DHPG responses, obtained either 30–40 or 50–60 min post-break-in, and found that they were indistinguishable (p>0.1, Figs. 5b, 5d). Hence, gradual diminution in the eCB_mGluR_ response depended on the presence of both the DAGL inhibitor and repeated DHPG stimulation, and could not be explained simply by the duration of the inhibitor application.

The decline in eCB_mGluR_ just described might reflect use-dependent depletion of a pool of 2-AG. Two predictions would follow from this hypothesis: 1) evoking eCB_mGluR_ with a low concentration of DHPG should cause less of, or a slower onset of, a reduction in the DHPG effect in the presence of a DAGL inhibitor, and 2) inhibiting 2-AG synthesis by blocking another major synthetic enzyme in this pathway, phospholipase C_β_ [19], should also give rise to a use-dependent decline in eCB_mGluR_.

In experiments thus far, we used 50 µM DHPG, which is a high concentration. To test the prediction that, in the presence of a DAGL inhibitor, weaker stimulation of mGluRs would induce less decline in eCB_mGluR_, we used 10 µM DHPG, with 20 µM OMDM-188 in the internal solution. In this case, we did not observe significant reduction in eCB_mGluR_, even with four applications of DHPG given to the same cell (1^st^ DHPG: to 69.9±8.3% of baseline; 2^nd^ DHPG: to 72.9±7.5% of baseline; 3^rd^ DHPG: to 70.1±9.9% of baseline; 4^th^ DHPG: to 74.6±9.2% of baseline; n = 5, p>0.1, data not shown). Hence, the declines in eCB_mGluR_ seen with the higher DHPG concentration were not caused simply by repetitive activation of mGluRs.

**Figure 6:**
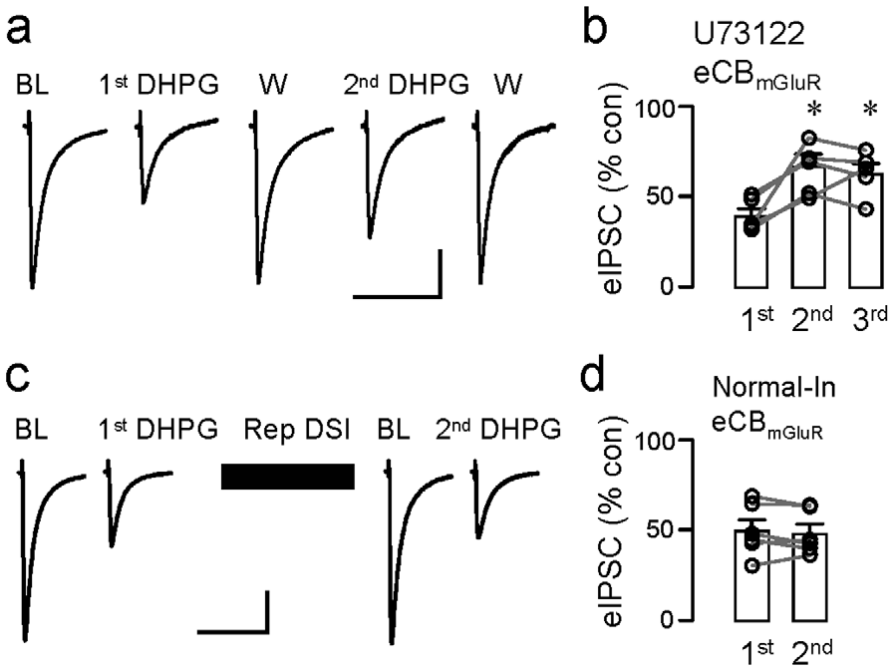
Regulation of eCB_mGluR_. (a) Sample eIPSCs (each trace is the mean of 3) from the same U73122-loaded cell in the indicated conditions (U73122, 6 µM, also present in the bath). BL denotes the baseline response, and W, the response obtained after washing out DHPG. DHPG was applied 3 times at 20-min intervals starting ∼15–20 min after break-in. (b) Group data for experiments in (a); ^*^p<0.01, one way repeated ANOVA; Scale: 20 ms/200 pA. (c) Sample eIPSCs (each trace is the mean of 3) response to 50 µM DHPG before and after repetitive DSI stimulation (1-s depolarizing steps were given at 12-s intervals continuously for 4 min). (d) Group data showing that eCB_mGluR_ was not affected by repetitive DSI stimulation. Scale: 20 ms/200 pA.

In testing the second prediction, we found that both DSI and eCB_mGluR_ were induced in the presence of the PLC inhibitor, U73122 (6 µM), however, when elicited repeatedly, eCB_mGluR_ suppression of eIPSCs diminished (1^st^ DHPG: eIPSCs were reduced to 40.1±3.8% of baseline; 2^nd^ DHPG: to 67.3±6.9% of baseline; 3^rd^ DHPG: to 62.1±5.8% of baseline; n = 5, p<0.01, Figs. 6a, 6b). As expected, since DSI is normal (though eCB_mGluR_ is abolished) in the PLC_β1_^−/−^ mouse [19], DSI was not altered by the PLC inhibitor (eIPSC reduction to 66.8±3.5% of baseline, n = 8).

If DSI and eCB_mGluR_ arise from the same source of 2-AG, then strong activation of one mechanism could alter the response of the other. Because strong stimulation of mGluRs with bath-applied DHPG has persistent effects on eIPSCs and DSI [14], [20] that could confound interpretation, we tested this prediction by determining if repetitive elicitation of DSI would affect eCB_mGluR_. Pipettes contained normal intracellular solution. We used two 4-min applications of 50 µM DHPG separated by a 4-min period during which DSI was elicited with 1-s depolarizing steps given at 12-s intervals. The 12-s interval is too short to permit full recovery from each DSI episode, with the result that eIPSCs are continually suppressed by the DSI mechanism for the period of stimulation (cf, [20]). As we have reported, an even longer period of repetitive elicitation does not persistently diminish DSI [20], nevertheless, it was possible that repetitive DSI stimulation could reduce the magnitude of the subsequent eCB_mGluR_ interval if a common 2-AG pool was being tapped. Nevertheless, we found no evidence that this occurred. The eIPSCs were suppressed to 49.8±5.8% of baseline by 50 µM DHPG before the repetitive DSI stimulation and to 48.4±4.8% of baseline afterwards; n = 6, p>0.1; Figs. 6c, 6d). We also tested the possibility that repetitive DSI stimulation might somehow alter eCB_mGluR_ if DSI expression was first blocked by OMDM-188. Again, after a 4-min period of repetitive DSI, 50 µM DHPG induced an eIPSC suppression to 48.6±5.2% of baseline, not significantly different from the 1^st^ DHPG responses obtained either 10–20 or 30–40 min post break-in, p>0.5 (data not shown).

## Discussion

During a previous investigation [20], we noticed differences in the efficacy of THL on DSI or eCB_mGluR_, however, in view of the non-specific effects of THL, no definite conclusions could be drawn. Moreover, it was unclear if both DSI and eCB_mGluR_ were mediated by the same eCB. In showing that DAGLα, and by implication 2-AG, are involved in both processes, the recent studies on DAGLα^−/−^ mice [7], [8] prompted an examination of whether or not the same sources of 2-AG mediate eCB responses evoked by different stimuli. The hypothesis that a unitary pool of DAGLα supplies 2-AG for DSI and eCB_mGluR_ predicts they should be similarly affected by pharmacological inhibitors of DAGLα. Using different inhibitors and modes of drug application, we observed marked quantitative distinctions between the responses produced by DSI and eCB_mGluR_. In particular: 1) eCB_mGluR_ is much less sensitive to block by DAGL-inhibitors than is DSI, and 2) repetitive activation of the eCB_mGluR_ system enhanced the effect of DAGL inhibition, whereas such use-dependence was not a feature of the block of DSI.

The differences in sensitivity to the DAGL inhibitors were obvious even when both responses were recorded in the same cell, ruling out systematic differences between experiments. Differences in inhibitor- enzyme interactions are also ruled out, as DAGLα mediates both responses. A reasonable interpretation is that different pools of DAGLα provide 2-AG in the two cases. The hypothetical pools would not simply represent differences in spatial localization along the pyramidal cell: with external application the inhibitors have equal access to the surface of the cells, but had only slight effects on eCB_mGluR_, despite abolishing DSI. A plausible explanation for the differing efficacy of internal and external application on eCB_mGluR_ is that the DAGLα responsible for eCB_mGluR_ is much less accessible to externally applied inhibitor.

The suggestion of different pools of DAGLα is in good agreement with previous observations. For example, the DAGLα involved in eCB_mGluR_ is found in dendrites apposed to glutamate releasing nerve terminals [21], [22], [23]. In contrast, DAGLα has not been reported near perisomatic GABAergic synapses like those we have studied [21], [22]. Since eCBs can spread longitudinally along cell structures for only ∼≤10 µm [24], 2-AG produced by DAGLα near excitatory synapses, which are located on CA1 pyramidal cell dendrites >50 µm from the somata, is most unlikely to account for DSI. While the failure to have detected DAGLα in pyramidal cell somata may reflect technical limitations in available morphological tools, it does highlight the possibility that different parts of a cell employ different pools of DAGLα for generating eCBs. We also note, however, that while this seems to be a parsimonious proposal, the lack of identification of the DAGLα responsible for DSI means that other possibilities are not ruled out. For example, our data would be compatible with differences in DAG (rather than DAGLα) pools. DAG can be produced by several mechanisms besides PLC [5]. We confirm the 2-AG produced by mGluRs is dependent on PLC_β_, but knocking out [19], or inhibiting [20] PLC_β_ does not affect hippocampal DSI. Since DSI is dependent on DAGLα, and therefore probably on 2-AG, it could be mediated by a source of DAG that is distinct from that underlying eCB_mGluR_. Interestingly, use-dependence of eCB_mGluR_ reduction was seen when PLC, rather than DAGL, was inhibited, supporting the concept that the DAGLα-PLC pathway is upstream of the depletable source of 2-AG for eCB_mGluR_.

Attempts to use DAGLα inhibitors to probe the eCB system have produced controversial results [18], [20], [25], [26]. We had observed that external, though not internal, THL application affected DSI [26], and Min et al. [25] arrived at conclusions that are diametrically opposed to our present observations. Probably the use of higher concentrations of the inhibitors, longer application times, and repetitive activation of the 2-AG-dependent responses are the primary explanations for the reported variability. In particular, difficulties in bath- or internally-applying these lipophilic agents to cells in brain slices could account for the requirement for higher concentrations, especially with intracellular techniques, because restricted efflux from whole-pipettes can result from partial electrode occlusion or adherence of the drugs to pipette glass. Nevertheless, the possibility of non-specific effects must also be kept in mind.

The use-dependence of the reduction in eCB_mGluR_ is puzzling, and while direct evidence is not available, one speculative scenario is intriguing: decoupling of 2-AG synthesis and release could partly account for the data. For example, if the pool of DAGLα-dependent 2-AG that is present in unstimulated cells [4], [7], [8] could be mobilized by mGluR activation, its release would not be closely tied to DAGLα stimulation. Release from such a pool could persist after DAGLα was inhibited, decreasing in a use-dependent way as the preformed 2-AG pool diminished. If such decoupling were greater for eCB_mGluR_ than DSI, it could also account for their different sensitivities to DAGL inhibitors. Finally, 2-AG release from an existing pool could explain why knocking out PLC_β_ abolishes eCB_mGluR_ [19], while a potent PLC inhibitor was ineffective [20], [26] (unless eCB_mGluR_ is repetitively elicited, Fig. 6). Thus, our present data is consistent with, and extends, previous proposals [26], [27], [28].

Factors that could be responsible for maintaining the hypothetically distinct DAGLα (or DAG) pools are unknown. Physical separation of protein components of the biosynthetic cascades, perhaps sequestration on lipid rafts [29] or other factors related to the heterogeneity of the lipid bilayer could be involved [28]. In this context, it may also be worth noting that DAGLα^−/−^ mice also showed a reduction of ∼50% in the levels of anandamide [7], [8]. Therefore, although the greater reduction in 2-AG supports the concept that 2-AG is the eCB that mediates many phasic CB1R-dependent responses, the possibility cannot be eliminated that anandamide might play a contributory role in some cases.

The model of 2-AG release from a pre-existing pool differs from the conventional “on-demand” model of eCB production in which synthesis and release are necessarily tightly coupled to each other. The hypothesis may account in part for some of the multiple mechanisms of eCB responses [20], [26], and may help reconcile the negative pharmacological reports with DAGL inhibitors, e.g. [25], with data from the DAGL^−/−^ studies [7], [8]. The hypothesis would also be consistent with evidence that 2-AG release is a regulated step, perhaps involving the eCB transporter [30], [31]. Testing this idea and investigating the subcellular distribution and regulation of DAGL in general will be important future tasks.

## Methods

### Preparation of slices

All experimental protocols were reviewed and approved by the University of Maryland School of Medicine IACUC (IACUC approval #0609001), and all animal handling was conducted in accordance with national and international guidelines. The number of animals used was minimized, and all necessary precautions were taken to mitigate pain or suffering.

Hippocampal slices were obtained from 4- to 6-week-old male Sprague-Dawley rats. After rats were sedated with isoflurane and decapitated, the hippocampi were removed and 400-µm-thick slices were cut on a Vibratome (model VT1200s, Leica Microsystems, Inc., Bannockburn, IL) in an ice-cold extracellular recording solution. Slices were stored in a holding chamber on filter paper at the interface of this solution and a moist, oxygenated atmosphere at room temperature for ≥1h before transfer to the recording chamber (RC-27L, Warner Instruments, CT) and warmed to 30–31°C. The extracellular solution contained (mM): 120 NaCl, 3 KCl, 2.5 CaCl_2_, 2 MgSO_4_, 1 NaH_2_PO_4_, 25 NaHCO_3_, and 20 glucose, and was bubbled with 95%O_2_, 5%CO_2_ (pH 7.4).

### Electrophysiology

Whole-cell voltage-clamp recordings of CA1 pyramidal cells were made using the blind patch method. Pipettes were pulled from thin walled glass capillaries (1.5 O.D., World Precision Instruments, Sarasota, FL). Electrode resistances in the bath were 3–6 MΩ with internal solution containing (mM): 90 CsCH_3_SO_4_, 1 MgCl_2_, 50 CsCl, 2 MgATP, 0.2 Cs_4_-BAPTA, 10 HEPES, 0.3 Tris GTP and 5 QX314. If the series resistances, which was checked by -2 mV voltage steps throughout experiments, changed >20%, the data were discarded. The holding potential was -70 mV in all experiments. Monosynaptic eIPSCs were elicited by 100-µs-long extracellular stimuli delivered at 0.25 Hz with concentric bipolar stimulating electrodes placed in s. radiatum. NBQX (10 µM) and D-AP5 (20 µM) were present in all experiments to block glutamatergic EPSCs. Slices were pretreated in the holding chamber with the irreversible P/Q-type voltage-gated Ca^2+^- channel toxin, ω-agatoxin GVIA (agatoxin, 300 nM) for ≥1 h to reduce the contribution of eCB-insensitive eIPSCs in all experiments [32]. Data were collected with an Axopatch 1C amplifier (Molecular Devices, Sunnyvale, CA), filtered at 1 kHz and digitized at 5 kHz using a Digidata 1200 (Molecular Devices) and Clampex 8 software (Molecular Devices).

The bath solution was oxygenated with 95% O_2_/5% CO_2_ gas, and perfused continuously through the recording chamber at ∼1 ml/min. For external applications, slices were preincubated for >40 min with OMDM-188, THL, OMDM-169, DMSO, or ethanol, and the drug was also present in the bath solution throughout the recording. The final concentration of the solvent, DMSO or ethanol, was 0.05% (v/v) or less for both OMDM-188 and THL. JZL184 was obtained from Cayman Chemical, OMDM-188 and OMDM-169 was synthesized by Giorgio Ortar and Enrico Morera, and all other chemicals were purchased from Sigma (St. Louis, MO).

### Data analysis

To measure DSI, we evoked IPSCs at 4-s intervals and depolarized the postsynaptic cell to 0 mV for 1- 5 s at 90-s intervals. The magnitude of DSI was calculated as follows: DSI (%)  = 100× [1 - (mean of 4 IPSCs after depolarization/mean of 5 IPSCs before depolarization)]. Values of 2 - 3 DSI trials were averaged for a given condition. The decay time constant of DSI (τ_decay_) was determined by fitting the data with a single-exponential decay function in SigmaPlot 10.0. Two-tailed paired t-tests were used whenever appropriate; otherwise unpaired t-tests were used for single comparisons. Statistical tests among groups were done with one-way ANOVA. For comparison of results from repeated DHPG applications, we used one-way repeated ANOVA. The significance level for all tests was p<0.05 (*). Group means ± SEMs are shown for display purposes. For comparison of cumulative distributions, we used the Kolmogorov-Smirnov (K-S) test, available at http://www.physics.csbsju.edu/stats/KS-test.n.plot_form.html.
